# Norovirus prevalence and estimated viral load in symptomatic and asymptomatic children from rural communities of Vhembe district, South Africa

**DOI:** 10.1016/j.jcv.2016.09.005

**Published:** 2016-11

**Authors:** Jean Pierre Kabue, Emma Meader, Paul R. Hunter, Natasha Potgieter

**Affiliations:** a Department of Microbiology, School of Mathematical and Natural Sciences, University of Venda, Thohoyandou, RSA, South Africa; b School of Medicine, Health Policy and Practice, University of East Anglia, Norwich, UK; c Department of Environmental Health, Tshwane University of Technology, Pretoria, RSA, South Africa; d Dean, School of Mathematical and Natural Sciences, University of Venda, Thohoyandou, RSA, South Africa

**Keywords:** PHC, public health care, IC, internal control, ROC, receiver operating characteristic, Inv, inverse, RSA, Republic of South Africa, UK, United Kingdom, Norovirus, Symptomatic, Asymptomatic, Rural, Genogroup, Viral load

## Abstract

**Background:**

Human Norovirus (NoV) is recognized as a major etiological agent of sporadic acute gastroenteritis worldwide.

**Objectives:**

This study describes the clinical features associated with Human NoV occurrence in children and determines the prevalence and estimated viral burden of NoV in symptomatic and asymptomatic children in rural South Africa.

**Study design:**

Between July 2014 and April 2015, outpatient children under 5 years of age from rural communities of Vhembe district, South Africa, were enrolled for the study. A total of 303 stool specimens were collected from those with diarrhea (n = 253) and without (n = 50) diarrhea. NoVs were identified using real-time one-step RT-PCR.

**Results:**

One hundred and four (41.1%) NoVs were detected (62[59.6%] GII, 16[15.4%] GI, and 26[25%] mixed GI/GII) in cases and 18 (36%) including 9(50%) GII, 2(11.1%) GI and 7(38.9%) mixed GI/GII in controls. NoV detection rates in symptomatic and asymptomatic children (OR = 1.24; 95% CI 0.66–2.33) were not significantly different. Comparison of the median C_T_ values for NoV in symptomatic and asymptomatic children revealed significant statistical difference of estimated GII viral load from both groups, with a much higher viral burden in symptomatic children.

**Conclusions:**

Though not proven predictive of diarrhea disease in this study, the high detection rate of NoV reflects the substantial exposure of children from rural communities to enteric pathogens possibly due to poor sanitation and hygiene practices. The results suggest that the difference between asymptomatic and symptomatic children with NoV may be at the level of the viral load of NoV genogroups involved.

## Highlights

• NoV detection rates in cases and controls from children in Rural South Africa were not significantly different.
• Estimated GII viral load significantly higher in symptomatic than in asymptomatic children.
• First report on the difference between cases and controls with NoV in rural African population related to the viral load of NoV genogroups.

## 1 Background

More than 70% of African people who live in poverty, reside in rural areas [1], [2]. Subsequently illiteracy, malnutrition, inadequate water supplies and poor sanitation, as well as poor health and hygiene practices, affect a large proportion of rural communities in the African continent.

With the considerable decline of rotavirus-associated diarrhea in countries that have introduced rotavirus vaccines, NoV is increasingly recognized as a leading cause of acute gastroenteritis [3], [4].

The symptoms associated with NoV infection, which manifest after an incubation period of 1–2 days (95% CI 1.1–1.2 days) [5], are typically self-limiting, characterised by nausea, vomiting, abdominal pain and non-bloody diarrhea (4–8 stools per day). The duration of NoV illness is typically 12–72 h [6] but the illness can be prolonged (and severe) in the very young or old, and immunocompromised persons [7], [8]. However, reports have revealed that not all individuals develop symptoms and a significant proportion remains asymptomatic after NoV infections [9], [10], [11]. Several studies have suggested that the semi-quantitative measure of real-time RT-PCR as a proxy measure of fecal viral load using threshold cycles (C_T_) value may distinguish between asymptomatic viral shedding from clinically relevant disease [12], [13], [14].

Studies have shown that children from poor communities in developing countries with poor standards of hygiene, including unsafe disposal of faeces and the use of contaminated water supplies can facilitate the transmission of NoV [15], [16], [17]. Nevertheless most of the NoV studies in Africa have been carried out in urban settings, likely due to the lack of laboratory capacity for Human NoV detection in rural settings [18]. In South Africa, little has been reported on the prevalence and circulating NoV genotypes across the country [18], [19], [20].

## 2 Objectives

To determine the prevalence of NoVs in asymptomatic and symptomatic children in rural communities of Vhembe district/South Africa and to compare the differences in viral burden as suggested by the RT-PCR C_T_ value.

## 3 Study design

This study was a cross-sectional, clinic-based investigation of out-patients, conducted from July 2014 to April 2015. Stool samples were randomly collected at different clinics situated within the rural communities of Vhembe District in Limpopo Province, South Africa. In South Africa, most cases of intestinal gastroenteritis are seen by the PHC centres (clinics) situated in the rural communities and only the severe cases (with dehydration) are directed by the clinic nurses to the hospitals. A total of 40 clinics were designated sampling sites for this study. Samples were transported to the University of Venda Microbiology laboratory and tested for NoV by RT-PCR.

### 3.1 Clinical samples

#### 3.1.1 Informed and ethical consent

The study protocol and consent procedures were approved by the Ethics committees of the Department of Health in the Limpopo Province (Ref. 4/2/2) and University of Venda (Ref. SMNS/13/MBY/0212). Written, informed consent was given by the parent or guardian of the child before stool sample collection.

#### 3.1.2 Capture forms for data collection

\After consent was given, personal details (date of birth, sex, date of diarrhea onset) as well as clinical data such as presence of fever, vomiting, abdominal pain or dehydration were collected. The consistency of the stool (according to the Bristol stool chart) was documented. The parent employment status as well as the family living conditions such as the source of water, presence of livestock and toilet seat use was also recorded.

#### 3.1.3 Sample collection

One stool sample from each child under 5 years of age, who presented to the clinic with diarrhea, was collected by the clinic nurse and kept at + 4 °C. Diarrhea was defined as three or more episodes of watery stool in the previous 24 h [21]. Stool specimens were collected from clinics on a weekly basis, transported on ice to the laboratory within 6 h and stored at − 20 °C until tested.

A total of 253 stool samples from symptomatic cases were collected for this study. Stool samples from patients with bloody diarrhea were excluded.

Fifty stool samples from healthy controls (children under 5 years attending the clinic for routine immunization with no episodes of acute gastroenteritis in the previous 30 days) were also collected.

### 3.2 RNA extraction, NoV detection and characterisation

The Boom method was employed to extract NoV RNA as previously described [22]. The method is based on the lysing and nuclease inactivating properties of the chaotropic agent guanidinium thiocyanate, together with the nucleic acid-binding proprieties of silica particles.

- RIDA^©^ GENE NOROVIRUS I & II real-time RT-PCR (r-Biopharm AG, Darmstadt, Germany) kits were used to detect NoV from clinical samples in this study. This PCR assay offers qualitative detection and differentiation of NoV genogroup I and II in human stool samples according to the manufacturer and it is not thought to cross-react with other common enteric pathogens. RIDA gene kit can also detect GIV genogroup. The assay has 98% of sensitivity and specificity [23] and includes an internal control to monitor for extraction efficiency and amplification inhibition. The test is carried out in a one-step real-time RT-PCR format in which the reverse transcription of RNA is followed by the PCR in the same tube. The real-time PCR program was performed on a Corbett Research Rotor Gene 6000 with the following cycling conditions: Reverse transcription for 10 min at 58 °C; initial denaturation step for 1 min at 95 °C followed by 45 cycles of 95 °C for 15 s and 55 °C for 30 s with continuous fluorescence reading. Separate rooms were used for the pre- and post-amplification steps to minimise the risk of amplicon carry-over and contamination of samples.

Randomly selected stool RNA extracts, which tested NoV positive, were subjected to RT-PCR amplification using primers from previously published work, for the purpose of sequencing to confirm the detection results. The One step Ahead RT-PCR (QIAGEN) was used, utilising specific oligonucleotide primer sets GISKF/GISKR to amplify 330 bp of GI capsid fragment and GIISKF/GIISKR for 344 bp of GII capsid fragment as previously described [24]. The PCR products of the amplified fragments were directly purified with a master mix of ExoSAP (Nucleics, Australia). Using the same specific primers, the Sanger sequencing was performed on the ABI 3500XL Genetic Analyzer POP7™ (Thermo-Scientific).

The nucleotide sequences were compared with those of the reference strains available in the NCBI GenBank using BLAST tool available at http://www.ncbi.nlm.nih.gov/blast then analysed for their genotypes using Noronet typing tools [25] available at http://www.rivm.nlm/norovirus/typingtool

### 3.3 Statistical analyses

Data was initially recorded in Microsoft Excel. All analyses were done by STATA v13. Logistic regression of being NoV positive, using the following predictors: types of water sources, specific symptoms and whether or not the patient had watery stool, was calculated. Mann-Whitney U, Wilcoxon W, Z test and a *t*-test comparing C_T_ values in cases and controls were performed. Non-parametric receiver operating characteristic analyses to assess the association between C_T_ values and illness were also performed.

A P-value of < 0.05 was considered to be statistically significant.

## 4 Results

### 4.1 Study characteristics

From July 2014 to April 2015, a total of 303 fecal samples, including 253 specimens from cases and 50 from healthy controls, were collected and examined for NoV. The median age was 10 months (range 1–60 months) in the symptomatic group and the sex distribution was 53.4% (135/253) male, 46.6% (118/253) female. In the control group the median age was 13 months (range 1–55 months) and this cohort was comprised of 50% (25/50) male and 50% (25/50) female participants. The most common clinical features of the symptomatic children were with diarrhea only (reported in 56.1% [142/253]) and diarrhea with vomiting (24.9% [63/253]). The demographic profiles and clinical characteristics of study participant children are described in Table 1, Table 2.

### 4.2 Norovirus prevalence and characterisation

Of the 253 fecal samples from symptomatic children, 104 were positive for NoV (41.1%; 95%CI 35.0-47.4%). Of these positive samples 62 [59.6%] were GII only, 16 [15.4%] were GI, and 26 [25%] were GI/GII mixed in symptomatic children. Of 50 control samples 18 were positive for NoV (36.0%; 95%CI 22.9-50.8%) including 9 (50%) GII, 2 (11.1%) GI and 7 (38.9%) G/GII mixed. The prevalence of NoV was higher in cases (OR = 1.24; 95% CI 0.66–2.33) though this was not statistically significant. Looking at each genotype whether as single agent or in combination, GI was detected in 42 (17%) of cases and 9 (18%) of controls and GII in 88 (35%) of cases and 16(32%) of controls. These differences were also not statistically significant.

The highest detection rate of NoV, in case patients, was found in the age group of 13–24 months (47.7%, 31/65) (Table 1). NoVs were predominantly detected from children presenting with liquid stool (50%, 52/104) (Table 2). There is a suggestion that liquid stool is associated with NoV positivity, but this was not statistically significant (Odds Ratio = 1.58; 95% CI 0.98–2.5). Also, no risk factor has been found with NoVs genogroup as a predictor of symptomatic cases (Appendix S1).

As can be seen from Table 2 there is no difference in reported symptoms between case patients positive for NoV and case patients negative for NoV.

**Fig. 1:**
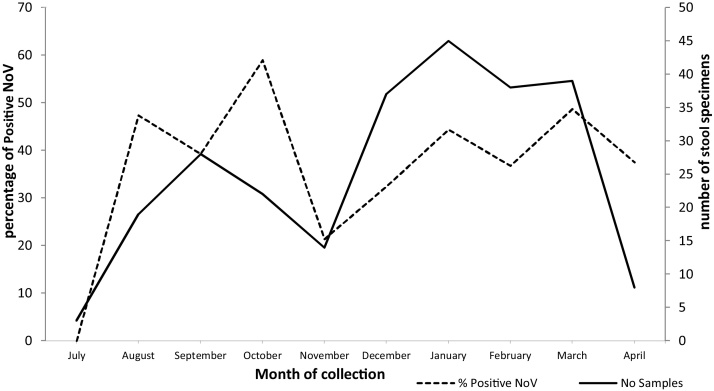
NoV detection rate by month between July 2014 and April 2015 in Children from rural communities of Vhembe district, South Africa.

Temporal distribution of NoV genogroups between July 2014 and April 2015 showed NoV detection every month throughout the study period with a possible peak in October 2014 (Fig. 1).

NoV-G2SKF/G2SKR amplicons of samples number 30, 45, 148 and NoV-G1SKF/G1SKR amplicons of samples number 139, 168, H011 were sequenced. A BLAST search confirmed that the sequenced samples were Human NoV (KJP-30C-Venda-2014, KJP-45C-Venda-2014, KJP-148C-Venda-2014, KJP-139C-Venda-2014, KJP-168C-Venda-2015, and KJP-H011C-Venda-2015). Noronet genotyping tool identified respectively the following Norovirus strains: GII.4 variant (n = 2), GII.14 (n = 1), GI.4 (n = 2) and GI.5 (n = 1) (Table 3).

### 4.3 Human NoV viral load in fecal specimens

**Fig. 2:**
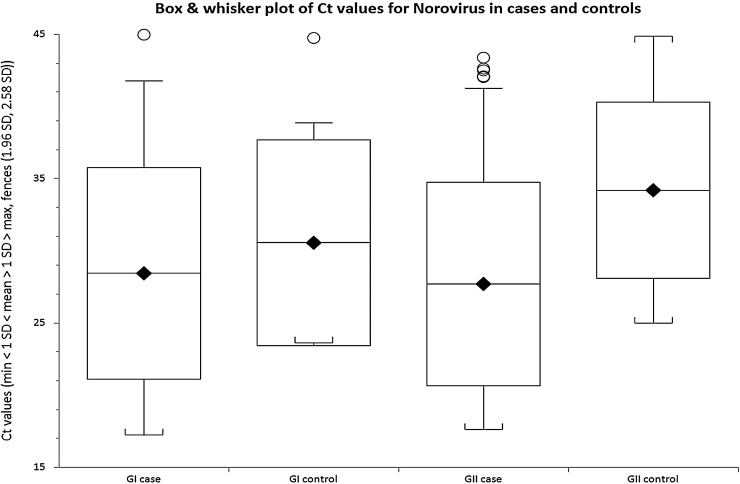
Comparison of Median C_T_ values of NoV in symptomatic and asymptomatic children from rural communities of Vhembe district, South Africa.

**Fig. 3:**
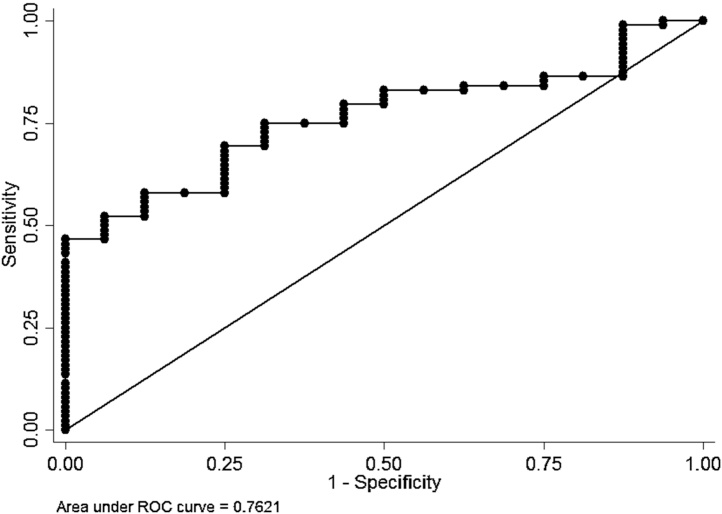
ROC curve for inv C_T_ values as predictors of diarrhea GII positive.

**Fig. 4:**
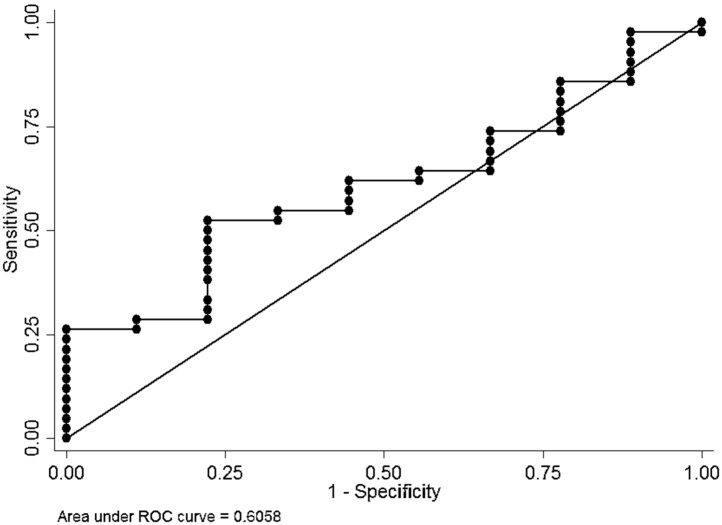
ROC curve for inv C_T_ values as predictors of diarrhea GI positive.

There was a considerable variation in NoV C_T_ values in positive samples from both symptomatic cases and asymptomatic controls (Fig. 2). The median C_T_ value of NoV GII genogroup in symptomatic was lower (27.02) than in asymptomatic children (34.59) and this was statistically significant (p = 0.0009 Kruskal-Wallis equality-of-populations rank test) (Fig. 2). However, there was no difference in median C_T_ value between symptomatic (28.06) and asymptomatic (27.58) participants for NoV GI (p = 0.32) (Fig. 2). The association between viral load, as estimated by C_T_ values, and illness was further investigated using non-parametric ROC analyses (Fig. 3, Fig. 4). For GII, it can be seen that there was a reasonable predictive power of C_T_ values, but not for GI.

Table 4 shows the sensitivity and specificity of using different C_T_ values for GI and GII as predictors of symptoms. It can be seen that although sensitivity of the GI and GII analyses are similar, the specificity for GI is much lower than for GII across all C_T_ values. Overall it would appear that the C_T_ values for GII adequately predict illness whereas this is not the case for GI. Specificity is poor, even for GII, except for C_T_ values below 20.

## 5 Discussion

The main objective of this study was to assess the NoV prevalence and compare the estimated viral load in asymptomatic and symptomatic children in rural communities of Vhembe district/South Africa. The results of this study revealed that the detection rate of NoV in symptomatic cases was high (41.1%, 104/253) but was not statistically different when compared to the controls (36%, 18/50). Evidence that NoV-positivity was more common in the symptomatic compared to the asymptomatic children was not established in this study. Furthermore NoV positive cases were not found to be predictors of symptoms.

Comparison of C_T_ values of NoV genogroups revealed a lower median C_T_ value (27.02) of NoV GII detected in symptomatic children, compared to that recorded for the asymptomatic children (34.59), and this was statistically significant. However, there was no significant difference in C_T_ values between NoV positive cases and controls for NoV GI genogroup.

Even though the prevalence of GII is roughly the same in cases and controls, the estimated viral load is higher in cases. We note that NoV GI genogroup, detected in both groups, did not exhibit the same trend suggesting that GI is not a cause of disease in the study population. The ROC analyses also revealed a considerable predictive power of C_T_ values for diarrhea GII positive, but not GI.

NoV-induced gastroenteritis has previously been associated with lower C_T_ values (implying higher viral loads), than asymptomatic infections in several studies [26], [12], [13], [27], [9], [14]. However, to our knowledge this is the first study reporting on the differences in estimated viral load of GII and GI NoV positive cases and controls. In real time PCR, C_T_ levels are used as a surrogate measurement of viral load in combination with standards of known quantities. In this study, the inhibition that may have affected the target C_T_ values, were monitored by the use of an internal control and all control C_T_ values were within the 30–32 cycle range.

The findings of the study are concordant with several studies that reported NoV GII as the predominant genogroup involved in clinical cases, and circulating in communities worldwide [28], [29], [30].

The observation that the prevalence of Human NoV excretion in stools is similar in both symptomatic and asymptomatic children has been previously reported and raises questions about its pathogenic role in Africa [31], [32], [33]. These findings also indicate that asymptomatic infections could be a source of NoV outbreaks. Similarly, Ayukekbong et al. [34] reported that in developing countries NoV infections are very common with comparable detection rates observed in diarrhea cases and controls. However in a cross-sectional study, it is easy to mis-classify substantial numbers of post-symptomatic infections as asymptomatic infections even when the controls are defined as absence of diarrhea symptoms in the preceding 4 weeks [35], [36], [37].

The high detection rate of NoV in children living in rural communities is likely to reflect their substantial exposure to enteric pathogens, probably as a result of poor sanitation and hygiene practices. Most of the children in the study population were from households with a very low income and poor living conditions, although comparable rates of NoV detection from outpatient children in rural communities and semi-urban settings have been reported previously in other developing countries such as Bolivia, China, Brazil and Mexico [38], [39], [40], [17].

The findings of this study are inconsistent with previous studies [38], [41] that found a substantial difference in the NoV detection rates of both groups. However these studies were carried out in semi-urban settings which are different from rural settings.

Children aged 13 to 24 months had the highest rates of NoV positivity relative to those of other age groups in this study. This finding is consistent with other studies of outpatient children in developing countries [42], [39], [43]. Young children between 13 and 24 months of age may have more opportunities to be exposed to NoV-infected environments that children of other age groups [39], coupled with the absence of toilet training.

One of the limitations of this study is the restricted number of stool specimens from healthy controls. Also we have not looked for other causes of gastroenteritis such as adenovirus, astrovirus or bacterial and parasitic causes. Though we have performed nucleotides sequencing of amplified capsid fragment on some samples at low virus concentration, the assay used in this study cannot help to differentiate Norovirus genotypes.

Our findings suggest that the difference between asymptomatic and symptomatic children in African populations may relate to the NoV viral load. The difference in estimated viral load of NoVs GI relative to GII observed in this study also supports the concept that transmissibility via the fecal-oral route and viral infectivity may be lower for GI than GII [44].

The study findings may have implications for the diagnosis of NoV disease and future vaccine development, which may only need to consider GII as the genogroup associated with diarrhea in the African population.

## Competing interests

None declared.

## Figures and Tables

**Table 1:** Demographic profile of NoV-positive in children under 5 years of age from rural communities of Vhembe district, South Africa.

	Case-patients		Controls	
	Total	No of positive (%)	Total	No of positive (%)
Detection rates (%)	253	104 (41,1)	50	18(36)

Age range (month)				
0−6	86	37(43,0)	9	6(66,7)
7–12	68	28(41,2)	15	4(26,7)
13–24	65	31(47,7)	16	5(31,2)
25–60	34	8(23,5)	10	3(30,0)

Gender				
Male	135	51(37,8)	25	10(40,0)
Female	118	53(44,9)	25	8(32,0)

**Table 2:** Clinical features of study participant children under 5 years of age from rural communities of Vhembe district, South Africa.

	Case-patients (*n* *=* *253*)						Controls (*n* *=* *50*)	
Parameter	*NoV*	*positive (%) *n* *=* *104**	*NoV*	*negative (%) *n* *=* *149**			*NoV*	*positive (%) *n* *=* *18**
Symptoms								
Diarrhoea only	59	(56.7)	83	(55.7)				
Diarrhoea+ other symptoms	45	(43.3)	66	(44.3)				

Other reported symptoms								
Dehydration	11	(10.6)	13	(8.7)				
Vomiting	28	(26.9)	35	(23.5)				
Anorexia	10	(9.6)	18	(12.1)				
Fever	20	(19.2)	28	(18.8)				

Type of stool								
Watery	52	(50.0)	58	(38.9)			0	
Formed	34	(32.7)	55	(36.9)			12	(66.7)
Soft	18	(17.3)	36	(24.2)			6	(33.3)

Interval^a^								
≤ 3days	87	(83.6)	127	(85.2)				
3 days	17	(16.4)	22	(14.8)				

a Between the onset of diarrhea and collection of stool.

**Table 3:** Genotyping results using BLAST and Noronet tool.

Sample number	Sequence name	Detection	Ct value	Confirmed Human Norovirus with BLAST	Sequence similarity	Genotype
30	KJP-30C-Venda-2014	GII	23.34	GII capsid	93–99%	GII.4 variant
45	KJP-45C-Venda-2014	GII	20.54	GII capsid	78–81%	GII.4 variant
148	KJP-148C-Venda-2014	GII	21.91	GII capsid	91–97%	GII.14
139	KJP-139C-Venda-2014	GI	23.94	GI capsid	87–90%	GI.4
168	KJP-168C-Venda-2015	GI	34.69	GI capsid	80–92%	GI.5
H011	KJP-H011C-Venda-2015	GI	32.69	GI capsid	94–99%	GI.4

**Table 4:** Sensitivities and specificities for using different C_T_ value cut off levels for predicting diarrhea in PCR positive NoV samples.

C_T_ cut off value	GI		GII	
	Sensitivity/%	Specificity/%	Sensitivity/%	Specificity/%
<40	95	11	92	13
<35	80	22	78	56
<30	68	33	70	69
<25	41	78	47	94
<20	15	100	8	100

## Appendix A Supplementary data

Table S1 Dataset for Analysis files 1 and 2.

Table S2 Logistic regression and other statistic tests.

## References

[1] UNICEF and WHO (2012). Progress on Drinking Water and Sanitation. http://www.unicef.org/wash/files/JMPreport.pdf.

[2] World Health Organization (WHO) and United Nations Development Programme (UNICEF) (2010). Progress on Sanitation and Drinking Water: 2010 Update. http://www.who.int/water_sanitation_health/publications/9789241563956/en/index.html.

[3] Hemming M., Rasanen S., Huhti L., Paloniemi M., Salminen M., Vesikari T. (2013). Major reduction of rotavirus, but not norovirus, gastroenteritis in children seen in hospital after the introduction of RotaTeq vaccine into the National Immunization Programme in Finland. Eur. J. Pediatr..

[4] Payne D.C., Vinje J., Szilagyi P.G. (2013). Norovirus and medically attended gastroenteritis in U.S. children. N. Eng. J. Med..

[5] Lee R.M., Lessler J., Lee R.A. (2013). Incubation periods of viral gastroenteritis: a systematic review. BMC Infect. Dis..

[6] Patel M.M., Hall A.J., Vinje J., Parashar U.D. (2009). Noroviruses: a comprehensive review. J. Clin. Virol..

[7] van Asten L., Siebenga J., van den Wijngaard C. (2011). Unspecified gastroenteritis illness and deaths in the elderly associated with norovirus epidemics. Epidemiology.

[8] Green K.Y., KDM, HPM (2007). Caliciviridae: the norovirus.

[9] Bareira D.M.P.G., Ferreira M.S., Fumian T.M. (2010). Viral load and genotypes of Noroviruses in symptomatic and asymptomatic children in Southeastern Brazil. J. Clin. Virol..

[10] Kindberg E., Akerlind B., Johnsen C. (2007). Host genetic resistance to symptomatic norovirus (GGII.4) infections in Denmark. J. Clin. Microbiol..

[11] Gallimore C.I., Cubitt D., du Plessis N., Gray J.J. (2004). Asymptomatic and symptomatic excretion of noroviruses during a hospital outbreak of gastroenteritis. J. Clin. Microbiol..

[12] Trang N.V., Choisy M., Nakagomi T. (2015). Determination of cut-off cycle threshold values in routine RT-PCR assays to assist differential diagnosis of norovirus in children hospitalized for acute gastroenteritis. Epidemiol. Infect..

[13] Elfving K., Andersson M., Msellem M.I. (2014). Real-time PCR threshold cycle cutoffs help to identify agents causing acute childhood diarrhea in zanzibar. J. Clin. Microbiol..

[14] Phillips G., Ben Lopman B., Tam C.C., Iturriza-Gomara M., Brown D., Gray J. (2009). Diagnosing norovirus-associated infectious intestinal disease using viral load. BMC Infect. Dis..

[15] Mattioli M.C., Pickering A.J., Gilsdorf R.J., Davis J., Boehm A.B. (2013). Hands and water as vectors of diarrheal pathogens in Bagamoyo, Tanzania. Environ. Sci. Technol..

[16] UNICEF (2012). Pneumonia and Diarrhoea: Tackling the Deadliest Diseases for the World’s Poorest Children. http://www.childinfo.org/files/Pneumonia_Diarrhoea_2012.pdf.

[17] García C.C., DuPont H., Long K.Z., Santos J.I. (2006). Asymptomatic norovirus infection in mexican children. J. Clin. Microbiol..

[18] Kabue J.P., Meader E., Hunter P.R., Potgieter N. (2016). Human Norovirus prevalence in Africa: a review of studies from 1990 to 2013. Trop. Med. Int. Health.

[19] Platts-Mills J.A., Babji S., Bodhidatta L. (2015). Pathogen-specifi c burdens of community diarrhoea in developing countries: a multisite birth cohort study (MAL-ED. Lancet Glob Health.

[20] Mans J., de Villiers J.C., du Plessis N.M., Avenant T., Taylor M.B. (2010). Emerging norovirus GII.4 2008 variant detected in hospitalised paediatric patients in South Africa. J. Clin. Virol..

[21] World Health Organization (WHO) (2005). Treatment of Diarrhea: a Manual for Physicians and Senior Health Workers. http://whqlibdoc.who.int/publications.

[22] Boom R., Sol M.M.M., Salimans C.L. (1990). Jansen PME. Wertheimvan Dillen, and J. van der Noordaa. Rapid and simple method for purification of nucleic acids. J. Clin. Microbiol..

[23] Dunbar N.L., Bruggink L.D., Marshall J.A. (2014). Evaluation of the RIDAGENE real-time PCR assay for the detection of GI and GII norovirus. Diagn. Microbiol. Infect. Dis..

[24] Kojima S., Kageyama T., Fukushi S. (2012). Genogroup-specific PCR primers for detection of Norwalk-like viruses. J. Virol. Methods.

[25] Kroneman A., Vennema H., Deforche K. (2011). An automated genotyping tool for enteroviruses and noroviruses. J. Clin. Virol..

[26] Ballard S.B., Saito M., Mirelman A.J., Bern C., Gilman R.H. (2015). Tropical and travel-associated norovirus: current concepts. Curr. Opin. Infect. Dis..

[27] Saito M., Goel-Apaza S., Espetia S. (2014). Multiple norovirus infections in a birth cohort in a Peruvian periurban community. Clin. Infect. Dis..

[28] Hoa Tran T.N., Trainor E., Nakagomi T., Cunliffe N.A., Nakagomi O. (2013). Molecular epidemiology of noroviruses associated with acute sporadic gastroenteritis in children: global distribution of genogroups, genotypes and GII.4 variants. J. Clin. Virol..

[29] Siebenga J.J., Vennema H., Zheng D.P., Vinje J., Lee B.E. (2009). Norovirus illness is a global problem: emergence and spread of norovirus GII.4 variants, 2001–2007. J. Infect. Dis..

[30] Patel M.M., Widdowson M.A., Glass R.I., Akazawa K., Vinje J., Parashar U.D. (2008). Systematic literature review of role of Noroviruses in sporadic gastroenteritis. Emerg. Infect. Dis..

[31] Huynen P., Mauroy A., Martin C. (2013). Molecular epidemiology of norovirus infections in symptomatic and asymptomatic children from Bobo Dioulasso, Burkina Faso. J. Clin. Virol..

[32] Trainor E., Lopman B., Iturriza-Gomara M. (2013). Detection and molecular characterisation of noroviruses in hospitalised children in Malawi, 1997–2007. J. Med. Virol..

[33] Mattison K., Sebunya T.K., Shukla A., Noliwe L.N., Bidawid S. (2010). Molecular detection and characterization of noroviruses from children in Botswana. J. Med. Virol..

[34] Ayukekbong J.A., Mesumbe H.N., Oyero O.G., Lindh M., Bergström T. (2015). Role of Noroviruses as aetiological agents of diarrhoea in developing countries. J. Gen. Virol..

[35] Lopman B., Kang G. (2014). In praise of birth cohorts: norovirus infection, disease,and immunity. Clin. Infect. Dis..

[36] Milbrath M.O., Spiknall I.H., Zelner J.L., Moe C.L., Eisenberg J.N.S. (2013). Heterogeneity in Norovirus shedding duration affects community risk. Epidemiol. Infect..

[37] Atmar R.L., Opekun A.R., Gilger M.A. (2008). Norwalk virus shedding after experimental human infection. Emerg. Infect. Dis..

[38] McAtee C.L., Webman R., Gilman R.H. (2015). Burden of norovirus and rotavirus in children after rotavirus vaccine introduction, cochabamba, Bolivia. Am. J. Trop. Med. Hyg..

[39] Zou W., Cui D., Wang X., Guo H., Yao X., Jin M. (2015). Clinical characteristics and molecular epidemiology of noroviruses in outpatient children with acute gastroenteritis in huzhou of China. PLoS One.

[40] Ferreira M.S., Victoria M., Carvalho-Costa F.A. (2010). Surveillance of norovirus infections in the state of rio de janeiro, Brazil 2005–2008. J. Med. Virol..

[41] Moyo S.J., Hanevik K., Blomberg B. (2014). Genetic diversity of norovirus in hospitalized diarrhoeic children and symptomatic controls in Dar es Salaam, Tanzania. Infect. Genet. Evol..

[42] Shioda K., Kambhampati A., Halla A.J., Lopman B.A. (2015). Global age distribution of pediatric norovirus cases. Vaccine.

[43] Jia L., Qian Y., Zhang Y. (2014). Prevalence and genetic diversity of noroviruses in outpatient pediatric clinics in Beijing, China 2010–2012. Infect. Gen. Evol..

[44] Chan M.C.W., Sung J.J.Y., Lam R.K.Y. (2006). Fecal viral load and norovirus- associated gastroenteritis. Emerg. Infect. Dis..

