# Supplementary material for: Norovirus prevalence and estimated viral load in symptomatic and asymptomatic children from rural communities of Vhembe district, South Africa
Source: J Clin Virol. 2016 Nov;84:12–8. doi: 10.1016/j.jcv.2016.09.005 (PMC5099155; doi:10.1016/j.jcv.2016.09.005)
Supplement: Supplementary file 3 [file mmc3.docx]

**Logistic regression being positive for noro in case**

| **Variables in the Equation** | | | | | | | | | |
| --- | --- | --- | --- | --- | --- | --- | --- | --- | --- |
|  | | B | S.E. | Wald | df | Sig. | Odds Ratio | 95% C.I.for EXP(B) | |
|  |  |  |  |  |  |  |  | Lower | Upper |
| Step 1^a^ | Positive for Noro | .216 | .321 | .452 | 1 | .502 | 1.241 | .661 | 2.329 |
|  |  |  |  |  |  |  |  |  |  |
| a. Variable(s) entered on step 1: Positive. | | | | | | | | | |

**Logistic regression with different Genotypes**

| **Variables in the Equation** | | | | | | | | | |
| --- | --- | --- | --- | --- | --- | --- | --- | --- | --- |
|  | | B | S.E. | Wald | df | Sig. | Odds Ratio | 95% C.I.for EXP(B) | |
|  |  |  |  |  |  |  |  | Lower | Upper |
| Step 1^a^ | Neg |  |  | 1.820 | 3 | .611 |  |  |  |
|  | GI | .541 | .775 | .488 | 1 |  | 1.718 | .376 | 7.846 |
|  | GII | .392 | .406 | .929 | 1 |  | 1.479 | .667 | 3.282 |
|  | GI+GII | -.226 | .468 | .233 | 1 |  | .798 | .319 | 1.997 |
| a. Variable(s) entered on step 1: Results. | | | | | | | | | |

**Logistic regression with genotypes as predictors**

| **Variables in the Equation** | | | | | | | | | |
| --- | --- | --- | --- | --- | --- | --- | --- | --- | --- |
|  | | B | S.E. | Wald | df | Sig. | Odds Ratio | 95% C.I.for EXP(B) | |
|  |  |  |  |  |  |  |  | Lower | Upper |
| Step 1^a^ | GIPos | -.098 | .405 | .058 | 1 | .809 | .907 | .410 | 2.006 |
| a. Variable(s) entered on step 1: GIPos. | | | | | | | | | |
|  |  |  |  |  |  |  |  |  |  |

| **Variables in the Equation** | | | | | | | | | |
| --- | --- | --- | --- | --- | --- | --- | --- | --- | --- |
|  | | B | S.E. | Wald | df | Sig. | Odds Ratio | 95% C.I.for EXP(B) | |
|  |  |  |  |  |  |  |  | Lower | Upper |
| Step 1^a^ | GIIPos | .125 | .331 | .143 | 1 | .705 | 1.133 | .593 | 2.167 |
| a. Variable(s) entered on step 1: GIIPos. | | | | | | | | | |
|  |  |  |  |  |  |  |  |  |  |

**Mann-Whitney U**

| **Ranks** | | | | |
| --- | --- | --- | --- | --- |
|  | Case | N | Mean Rank | Sum of Ranks |
| cT GI | 0 | 9 | 30.44 | 274.00 |
|  | 1 | 42 | 25.05 | 1052.00 |
|  | Total | 51 |  |  |
| cT GII | 0 | 16 | 75.56 | 1209.00 |
|  | 1 | 88 | 48.31 | 4251.00 |
|  | Total | 104 |  |  |

| **Test Statistics^a^** | | |
| --- | --- | --- |
|  | cT GI | cT GII |
| Mann-Whitney U | 149.000 | 335.000 |
| Wilcoxon W | 1052.000 | 4251.000 |
| Z | -.988 | -3.324 |
| Asymp. Sig. (2-tailed) | .323 | .001 |
| a. Grouping Variable: Case | | |
| b. Not corrected for ties. | | |

**t test compaing ct values in cases and control**

| **Group Statistics** | | | | | |
| --- | --- | --- | --- | --- | --- |
|  | Case | N | Mean | Std. Deviation | Std. Error Mean |
| cT GI | 0 | 9 | 30.5533 | 7.12466 | 2.37489 |
|  | 1 | 42 | 28.0669 | 7.39289 | 1.14075 |
| cT GII | 0 | 16 | 34.1925 | 6.10478 | 1.52619 |
|  | 1 | 88 | 27.7032 | 7.04411 | .75090 |

| **Independent Samples Test** | | | | | | | | | | |
| --- | --- | --- | --- | --- | --- | --- | --- | --- | --- | --- |
|  | | Levene's Test for Equality of Variances | | t-test for Equality of Means | | | | | | |
|  |  | F | Sig. | t | df | Sig. (2-tailed) | Mean Difference | Std. Error Difference | 95% Confidence Interval of the Difference | |
|  |  |  |  |  |  |  |  |  | Lower | Upper |
| cT GI | Equal variances assumed | .151 | .699 | .921 | 49 | .362 | 2.48643 | 2.69968 | -2.93879 | 7.91164 |
|  | Equal variances not assumed |  |  | .944 | 11.993 | .364 | 2.48643 | 2.63465 | -3.25436 | 8.22722 |
| cT GII | Equal variances assumed | .342 | .560 | 3.453 | 102 | .001 | 6.48932 | 1.87907 | 2.76219 | 10.21645 |
|  | Equal variances not assumed |  |  | 3.815 | 22.910 | .001 | 6.48932 | 1.70092 | 2.96993 | 10.00870 |

**Logistic regression of predictors for specific symptoms**

Vomiting

| **Variables in the Equation** | | | | | | | | | |  |
| --- | --- | --- | --- | --- | --- | --- | --- | --- | --- | --- |
|  | | | B | S.E. | Wald | df | Sig. | Exp(B) | 95% C.I.for EXP(B) | |
|  |  |  |  |  |  |  |  |  | Lower | Upper |
| Step 1^a^ | | GIPos | .084 | .400 | .044 | 1 | .833 | 1.088 | .497 | 2.383 |
|  |  | GIIPos | .151 | .316 | .228 | 1 | .633 | 1.163 | .626 | 2.159 |
| a. Variable(s) entered on step 1: GIPos, GIIPos. | | | | | | | | | | |

Fever

| **Variables in the Equation** | | | | | | | | | |
| --- | --- | --- | --- | --- | --- | --- | --- | --- | --- |
|  | | B | S.E. | Wald | df | Sig. | Exp(B) | 95% C.I.for EXP(B) | |
|  |  |  |  |  |  |  |  | Lower | Upper |
| Step 1^a^ | GIPos | .226 | .431 | .274 | 1 | .601 | 1.253 | .538 | 2.919 |
|  | GIIPos | -.126 | .352 | .129 | 1 | .720 | .881 | .442 | 1.758 |
| a. Variable(s) entered on step 1: GIPos, GIIPos. | | | | | | | | | |

Dehydration

| **Variables in the Equation** | | | | | | | | | |
| --- | --- | --- | --- | --- | --- | --- | --- | --- | --- |
|  | | B | S.E. | Wald | df | Sig. | Exp(B) | 95% C.I.for EXP(B) | |
|  |  |  |  |  |  |  |  | Lower | Upper |
| Step 1^a^ | GIPos | .030 | .596 | .003 | 1 | .959 | 1.031 | .321 | 3.312 |
|  | GIIPos | -.077 | .470 | .027 | 1 | .869 | .926 | .368 | 2.326 |
| a. Variable(s) entered on step 1: GIPos, GIIPos. | | | | | | | | | |

Asthenia

| **Variables in the Equation** | | | | | | | | | |
| --- | --- | --- | --- | --- | --- | --- | --- | --- | --- |
|  | | B | S.E. | Wald | df | Sig. | Exp(B) | 95% C.I.for EXP(B) | |
|  |  |  |  |  |  |  |  | Lower | Upper |
| Step 1^a^ | GIPos | -.158 | .583 | .074 | 1 | .786 | .854 | .272 | 2.678 |
|  | GIIPos | -.394 | .449 | .770 | 1 | .380 | .675 | .280 | 1.625 |
|  | Constant | -1.864 | .234 | 63.239 | 1 | .000 | .155 |  |  |
| a. Variable(s) entered on step 1: GIPos, GIIPos. | | | | | | | | | |

**Logistic regression of cT value for GI on other symptoms**

| Vomiting       \| **Variables in the Equation** \| \| \| \| \| \| \| \| \| \| \| --- \| --- \| --- \| --- \| --- \| --- \| --- \| --- \| --- \| --- \| \|  \| \| B \| S.E. \| Wald \| df \| Sig. \| Odds Ratio \| 95% C.I.for EXP(B) \| \| \| Lower \| Upper \| \| Step 1^a^ \| cTGI \| -.071 \| .055 \| 1.690 \| 1 \| .194 \| .931 \| .837 \| 1.037 \| \| a. Variable(s) entered on step 1: cTGI. \| \| \| \| \| \| \| \| \| \| \|  \|  \|  \|  \|  \|  \|  \|  \|  \|  \| |  |  |  |  |  |  |  |  |  |
| --- | --- | --- | --- | --- | --- | --- | --- | --- | --- | --- | --- | --- | --- | --- | --- | --- | --- | --- | --- | --- | --- | --- | --- | --- | --- | --- | --- | --- | --- | --- | --- | --- | --- | --- | --- | --- | --- | --- | --- | --- | --- | --- | --- | --- | --- | --- | --- | --- | --- | --- | --- | --- | --- | --- | --- | --- | --- | --- | --- | --- | --- |

Fever

| **Variables in the Equation** | | | | | | | | | |
| --- | --- | --- | --- | --- | --- | --- | --- | --- | --- |
|  | | B | S.E. | Wald | df | Sig. | Odds Ratio | 95% C.I.for EXP(B) | |
|  |  |  |  |  |  |  |  | Lower | Upper |
| Step 1^a^ | cTGI | -.100 | .064 | 2.440 | 1 | .118 | .904 | .797 | 1.026 |
| a. Variable(s) entered on step 1: cTGI. | | | | | | | | | |
|  |  |  |  |  |  |  |  |  |  |

Dehydration

| **Variables in the Equation** | | | | | | | | | |
| --- | --- | --- | --- | --- | --- | --- | --- | --- | --- |
|  | | B | S.E. | Wald | df | Sig. | Odds Ratio | 95% C.I.for EXP(B) | |
|  |  |  |  |  |  |  |  | Lower | Upper |
| Step 1^a^ | cTGI | -.043 | .078 | .303 | 1 | .582 | .958 | .821 | 1.117 |
| a. Variable(s) entered on step 1: cTGI. | | | | | | | | | |
|  |  |  |  |  |  |  |  |  |  |

Asthenia

| **Variables in the Equation** | | | | | | | | | |
| --- | --- | --- | --- | --- | --- | --- | --- | --- | --- |
|  | | B | S.E. | Wald | df | Sig. | Odds Ratio | 95% C.I.for EXP(B) | |
|  |  |  |  |  |  |  |  | Lower | Upper |
| Step 1^a^ | cTGI | -.132 | .101 | 1.701 | 1 | .192 | .876 | .719 | 1.069 |
| a. Variable(s) entered on step 1: cTGI. | | | | | | | | | |
|  |  |  |  |  |  |  |  |  |  |

**Logistic regression of cT value for GII on other symptoms**

Vomiting

| **Variables in the Equation** | | | | | | | | | |
| --- | --- | --- | --- | --- | --- | --- | --- | --- | --- |
|  | | B | S.E. | Wald | df | Sig. | Odds Ratio | 95% C.I.for EXP(B) | |
|  |  |  |  |  |  |  |  | Lower | Upper |
| Step 1^a^ | cTGII | -.071 | .041 | 3.063 | 1 | .080 | .932 | .860 | 1.009 |
| a. Variable(s) entered on step 1: cTGII. | | | | | | | | | |
|  |  |  |  |  |  |  |  |  |  |

Fever

| **Variables in the Equation** | | | | | | | | | |
| --- | --- | --- | --- | --- | --- | --- | --- | --- | --- |
|  | | B | S.E. | Wald | df | Sig. | Odds Ratio | 95% C.I.for EXP(B) | |
|  |  |  |  |  |  |  |  | Lower | Upper |
| Step 1^a^ | cTGII | -.005 | .040 | .016 | 1 | .898 | .995 | .920 | 1.076 |
|  | Constant | -1.363 | 1.131 | 1.453 | 1 | .228 | .256 |  |  |
| a. Variable(s) entered on step 1: cTGII. | | | | | | | | | |

Dehydration

| **Variables in the Equation** | | | | | | | | | |
| --- | --- | --- | --- | --- | --- | --- | --- | --- | --- |
|  | | B | S.E. | Wald | df | Sig. | Odds Ratio | 95% C.I.for EXP(B) | |
|  |  |  |  |  |  |  |  | Lower | Upper |
| Step 1^a^ | cTGII | -.019 | .055 | .118 | 1 | .731 | .981 | .880 | 1.094 |
|  | Constant | -1.782 | 1.541 | 1.338 | 1 | .247 | .168 |  |  |
| a. Variable(s) entered on step 1: cTGII. | | | | | | | | | |

Asthenia

| **Variables in the Equation** | | | | | | | | | |
| --- | --- | --- | --- | --- | --- | --- | --- | --- | --- |
|  | | B | S.E. | Wald | df | Sig. | Exp(B) | 95% C.I.for EXP(B) | |
|  |  |  |  |  |  |  |  | Lower | Upper |
| Step 1^a^ | cTGII | .007 | .052 | .017 | 1 | .897 | 1.007 | .909 | 1.115 |
| a. Variable(s) entered on step 1: cTGII. | | | | | | | | | |
|  |  |  |  |  |  |  |  |  |  |

**Logistic regression of norovirus on whether or not watery stool**

| **Variables in the Equation** | | | | | | | | | |
| --- | --- | --- | --- | --- | --- | --- | --- | --- | --- |
|  | | B | S.E. | Wald | df | Sig. | Exp(B) | 95% C.I.for EXP(B) | |
|  |  |  |  |  |  |  |  | Lower | Upper |
| Step 1^a^ | Positive | .454 | .243 | 3.508 | 1 | .061 | 1.575 | .979 | 2.535 |
| a. Variable(s) entered on step 1: Positive. | | | | | | | | | |
|  |  |  |  |  |  |  |  |  |  |

| **Variables in the Equation** | | | | | | | | | |
| --- | --- | --- | --- | --- | --- | --- | --- | --- | --- |
|  | | B | S.E. | Wald | df | Sig. | Exp(B) | 95% C.I.for EXP(B) | |
|  |  |  |  |  |  |  |  | Lower | Upper |
| Step 1^a^ | GIPos | .589 | .324 | 3.300 | 1 | .069 | 1.802 | .955 | 3.403 |
|  | GIIPos | .130 | .263 | .246 | 1 | .620 | 1.139 | .681 | 1.906 |
|  | Constant | -.713 | .154 | 21.541 | 1 | .000 | .490 |  |  |
| a. Variable(s) entered on step 1: GIPos, GIIPos. | | | | | | | | | |
